# *SLC26A2*-Associated Diastrophic Dysplasia and rMED—Clinical Features in Affected Finnish Children and Review of the Literature

**DOI:** 10.3390/genes12050714

**Published:** 2021-05-11

**Authors:** Helmi Härkönen, Petra Loid, Outi Mäkitie

**Affiliations:** 1 Research Program for Clinical and Molecular Metabolism, Faculty of Medicine, University of Helsinki, 00014 Helsinki, Finland; helmi.harkonen@helsinki.fi (H.H.); petra.loid@helsinki.fi (P.L.); 2 Children’s Hospital and Pediatric Research Center, University of Helsinki and Helsinki University Hospital, 00029 Helsinki, Finland; 3 Folkhälsan Research Center, 00029 Helsinki, Finland

**Keywords:** SLC26A2, diastrophic dysplasia, multiple epiphyseal dysplasia, phenotype, skeletal dysplasia

## Abstract

Diastrophic dysplasia (DTD) is a rare osteochondrodysplasia characterized by short-limbed short stature and joint dysplasia. DTD is caused by mutations in *SLC26A2* and is particularly common in the Finnish population. However, the disease incidence in Finland and clinical features in affected individuals have not been recently explored. This registry-based study aimed to investigate the current incidence of DTD in Finland, characterize the national cohort of pediatric subjects with DTD and review the disease-related literature. Subjects with *SLC26A2*-related skeletal dysplasia, born between 2000 and 2020, were identified from the Skeletal dysplasia registry and from hospital patient registry and their clinical and molecular data were reviewed. Fourteen subjects were identified. Twelve of them were phenotypically classified as DTD and two, as recessive multiple epiphyseal dysplasia (rMED). From the subjects with available genetic data, 75% (9/12) were homozygous for the Finnish founder mutation c.-26+2T>C. Two subjects with rMED phenotype were compound heterozygous for p.Arg279Trp and p.Thr512Lys variants. The variable phenotypes in our cohort highlight the wide spectrum of clinical features, ranging from a very severe form of DTD to milder forms of DTD and rMED. The incidence of DTD in Finland has significantly decreased over the past decades, most likely due to increased prenatal diagnostics.

## 1. Introduction

Diastrophic dysplasia (DTD, MIM #222600) is a rare autosomal recessive chondrodysplasia caused by biallelic mutations in the sulfate transporter gene (*SLC26A2*), also known as diastrophic dysplasia sulfate transporter (*DTDST*) gene. The gene locates in distal chromosome 5q and encodes a sulfate transporter [1]. This transmembrane sulfate transporter is important for the uptake of sulfate into chondrocytes, in order to maintain adequate sulfation of proteoglycans and has an important role in endochondral bone formation. *SLC26A2* is expressed in several tissues with particularly high expression in developing and mature cartilage and epithelial tissues [2,3,4]. Mutations lead to impaired sulfate transportation through cell membranes, intracellular sulfate depletion and insufficiently sulfated proteoglycans [5]. The phenotypic severity of DTD has been related to residual sulfate uptake capacity and degree of proteoglycan under-sulfation [6]. Mutations in *SLC26A2* lead to a wide spectrum of both lethal and non-lethal skeletal dysplasia. The lethal conditions include achondrogenesis type 1B (ACG1B, MIM #600972) and atelosteogenesis type 2 (AO2, MIM #256050. The non-lethal conditions are diastrophic dysplasia (DTD) and recessive multiple epiphyseal dysplasia (rMED, MIM #226900) [7,8]. These disorders are inherited in an autosomal recessive manner; all heterozygous mutation carriers are asymptomatic. *SLC26A2* is the only gene linked to DTD [9].

DTD is found in almost all populations but is exceptionally common in the Finnish population. In 1990, about 160 subjects with DTD were known in Finland [10]. Over 250 subjects with DTD have been published elsewhere [11]. Approximately 90% of the individuals with DTD in Finland carry the Finnish founder mutation c.-26+2T>C, either in homozygosity or compounded with another *SLC26A2* mutation [12]. The p.Arg279Trp mutation is the most frequent mutation in non-Finnish populations; it causes rMED in homozygous state and usually DTD in compound heterozygous state [13]. Other common mutations are p.Cys653Ter and p.Arg178Ter [8,9,13].

Clinical features of DTD include short stature with limb shortening, contractures of large joints, spinal deformities, cleft palate, clubfoot, cystic swelling of the external ear and deformities of the hands. The median adult height is 136 cm for males and 129 cm for females [14]. DTD causes significant physical difficulties. Limb shortness and tightness of ligaments and joint capsules lead to limited joint mobility [9]. Flexion contractures of the knees, osteoarthrosis, scoliosis, foot deformities and obesity cause walking difficulties [15]. Physical difficulties in daily life affect the quality of life, together with economic and social challenges [16]. Clinical findings of the milder phenotype rMED are normal/mildly shortened stature, joint contractures, mild hand deformity, double-layered patellae and clubfoot [17,18,19,20]. The clinical features partly overlap with those of DTD.

Radiological findings in DTD include shortened long bones with metaphyseal flaring, flat epiphyses, kyphoscoliosis, cervical kyphosis, bowed radius and tibia, proximally situated “hitchhiker” thumb with shortness of the first metacarpal, brachydactyly and ulnar deviation of fingers. The diagnosis of DTD is made by a combination of clinical and radiological findings and confirmed with molecular genetic testing targeting the *SLC26A2* gene. Prenatal diagnosis can be performed by ultrasound and genetic testing [9,21].

There is currently no curative treatment for DTD. The affected individuals are mainly treated with physiotherapy and corrective orthopedic surgery [22,23,24,25]. However, recent animal studies have shown promising results from treatment with N-acetylcysteine (NAC), which acts as an intracellular source of sulfate. Dtd mice treated with NAC showed an increase in cartilage proteoglycan sulfation and improvement of the skeletal phenotype [26,27]. These findings suggest that NAC could have some potential for pre- and postnatal pharmacological treatment for subjects with DTD. Another study in mice found a novel therapeutic target in the lethal forms of *SLC26A2*-related chondrodysplasias by discovering overactive fibroblast growth factor 3 (Fgfr3) signaling in *slc26a2*^−/−^ mice. FGFR3 signaling is an important pathway in the regulation of chondrocyte growth. Suppression of Fgfr3 signaling by inhibition of Fgfr3 or phosphorylation of the downstream effectors improved cartilage growth in vitro and pathological characteristics of *slc26a2*^−/^^−^ newborn mice [28], suggesting that this could be another treatment target in DTD.

The incidence of DTD in Finland has not been explored recently. This study aimed to investigate the incidence of DTD in Finland during the recent years, clinically characterize a cohort of Finnish pediatric DTD patients, and review the current literature on this disorder.

## 2. Materials and Methods

This retrospective register study was carried out at Children’s Hospital, Helsinki University Hospital, Finland. This study is part of an ongoing research program investigating the epidemiological, genetic and clinical features of skeletal dysplasia in the Finnish population. The care of children with skeletal dysplasia has for several years been centralized to Helsinki University Hospital. In addition, several nationwide studies on DTD have been carried out in Helsinki over the years, and these data have been systematically collected in the Finnish Skeletal dysplasia registry. We reviewed the Skeletal dysplasia registry for all subjects with DTD born during years 1950–2020 and the hospital patient register for all those with *SLC26A2*-associated skeletal dysplasia born in 2000 or later. Clinical and molecular data were collected from hospital records for all subjects born in 2000–2020. Growth data were compared with Finnish growth standards [29]. Ethical approval for this study was obtained from the research ethics committee of the Hospital District of Helsinki and Uusimaa.

## 3. Results

### 3.1. Number of Finnish DTD Subjects Born in 1950–2020

**Figure 1:**
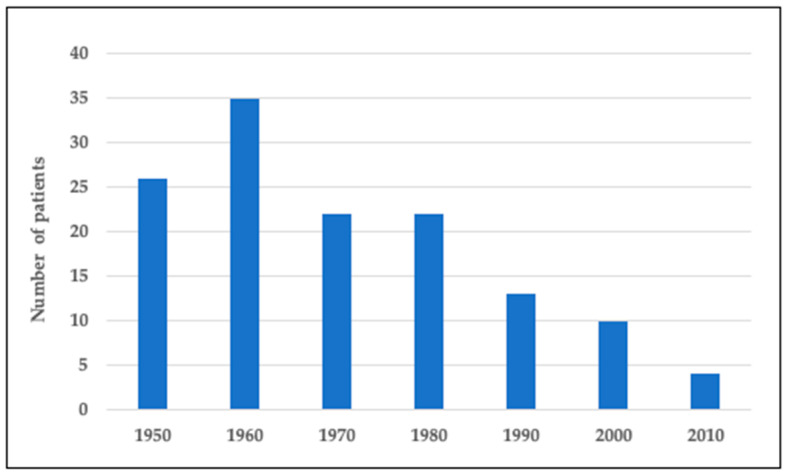
Number of individuals with DTD in Finland born in 1950–2020.

Figure 1 presents the number of Finnish subjects with DTD in the national skeletal dysplasia registry born in 1950–2020. We identified a total of 132 subjects. Our pediatric cohort, representing those born during 2000–2020, includes 14 individuals. There was a continuous downward trend in the number of children born with DTD over the years. While in 1950–1990 there were 22–35 new affected subjects born each decade, the number has since then diminished to on average 1 or no new subjects yearly (Figure 1).

### 3.2. Clinical and Molecular Description of the Pediatric Cohort

We identified 14 Finnish subjects in 13 families (seven males and seven females), born in 2000–2020, in the national skeletal dysplasia registry and hospital registry. In two of these individuals, a diagnosis of DTD was initially assumed, but the diagnosis was later confirmed to be rMED. One child with a very severe form of DTD deceased at the age of 8 days, while the more mildly affected sibling survived. The median age of the remaining 13 subjects was 13.5 years (range 5.3–20.8 years).

Table 1 presents the genetic information, clinical phenotype and length/height SDS at birth, age 1 year and age 5 years for the affected children in our cohort. Genetic information was available for 12 of them. Nine were homozygous for the Finnish *SLC26A2* founder mutation (c.-26+2T>C). One subject with a milder form of DTD was heterozygous for the Finnish founder mutation, but information about the second mutation was not available. However, the clinical presentation was typical for DTD. Two subjects with rMED phenotype were compound heterozygous for p.Arg279Trp and p.Thr512Lys variants; these subjects have been previously described [30,31].

Table 2 presents growth data at birth for the subjects with DTD and rMED. The children had usually a significant growth deficit already at birth. Median length SDS at birth was −4.1 for girls and −4.5 for boys with DTD, and −1.7 for the boys with rMED.

The growth failure progressed during the first year. Median length SDS at age 1 was −6.25 for girls and −6.5 for boys with DTD, and −1.45 for boys with rMED. The median decrease in length SDS from birth to age 1 was 1.8 for girls and 2.1 for boys with DTD. Median height SDS at age 5 was −5.5 for girls and −4.7 for boys with DTD. Median length-adjusted weight (weight-for-length; normal range −20%–+20%) at age 1 was 22% (range 10–49) for girls, 23% (range 20–33) for boys with DTD and 5.5% (range 4–7) for boys with rMED. Median weight-for-length at age 5 was 17% (range 12–25) for girls, 24% (range 14–53) for boys with DTD and 8% (range 7–9) for boys with rMED.

Median height SDS at last follow-up was −5.5 (range −4.5 to −6.8) for girls at median age 12 years (range 10–17 years), −4.1 (range −3.2 to −8.8) for boys with DTD at median age 13 years (range 9–16 years) and −2.9 (range −2.9 to −2.9) for boys with rMED at median age 12.5 years (range 5–20 years). Median weight-for-length at last follow-up was 35% (range 14–118) for girls, 53% (range 11–100) for boys with DTD, and 23.5% (range 7–40) for boys with rMED. These values indicate that the subjects with DTD have severe short stature, but the degree of growth failure varies considerably despite similar causative *SLC26A2* variants in most of the subjects.

Information about the time of diagnosis was available for 13 children; in 77% (10/13) DTD was suspected during pregnancy and in 23% (3/13) at birth. Ultrasound screening found abnormalities during pregnancy in ten subjects; in three, nuchal translucency ultrasound screening showed abnormalities and in all ten, structural ultrasound showed short limbs and suspicion of a developmental disorder. The diagnosis of DTD was prenatally confirmed from amniotic fluid sample in two subjects. In the remaining eight subjects, diagnosis of DTD was confirmed after birth. The suspicion of DTD at birth in the three subjects was based on clinical features like short limbs, clubfoot, cleft palate and hand abnormalities. Birth records were available for 13 subjects. Median duration of pregnancy was 39 + 1 weeks. Five subjects (36%) had respiratory insufficiency after birth; three of them were treated at the intensive care unit and one had severe pulmonary hypertension.

Table 3 presents the prevalence of clinical manifestations in our cohort. Hand abnormalities were present in all subjects; the most common findings were symphalangism of the fingers (n = 7), hitchhiker’s thumb/abduction of the thumb (n = 7), flexion tendency of the fingers (n = 6) and lack of proximal interphalangeal joints (n = 1). Cleft palate was present in 64%. Bipartite uvula was mentioned in four cases and one subject had a missing uvula. Three children had naevus flammeus in forehead, ten had small chin and five had auricular abnormalities (swelling/deformities).

Seven subjects had bilateral club foot deformity, one had unilateral club foot and two had metatarsus adductus deformity. The prevalence of knee problems was high; valgus deformity (86%), lateral position of patella (79%) and absence/laxity of the anterior cruciate ligament (ACL) (71%) were the main features. Five children (36%) had scoliosis. The children obtained their diagnosis of scoliosis at 0–14 years of age. One child had a rapidly progressing scoliosis at age 1 year. Pronounced lumbar lordosis was present in eight subjects (57%) and cervical kyphosis was present in 11 subjects (79%). The severity of cervical kyphosis varied from mild to severe. The kyphosis had resolved spontaneously in four children at the age of 2–7 years. One child required an operation and Glisson’s skull traction.

Two children with DTD had a diagnosis of early-onset osteoarthrosis; one had rapidly progressive arthrosis and ankylosis of the left hip at age 10 and the other had arthrosis of the lateral knee condyle at age 17. The most common surgeries performed were knee operations (n = 9), cleft palate (n = 8), club foot (n = 7) and achilles tenotomy (n = 6). Neck and spine operations were performed for one child.

### 3.3. Literature Review

Next, we performed a literature search in Pubmed to identify reports, written in English, describing non-Finnish *SLC26A2*-related DTD and rMED subjects and cohorts during 2000–2021. Table 4 presents the genetic and clinical findings of these previously reported subjects. The Finnish founder mutation c.-26+2T>C was rare and described in homozygosity only in one individual with DTD. Compounded with the most common *SLC26A2* variant, Arg279Trp it gave rise to rMED or an intermediate phenotype. The Arg279Trp in turn led to rMED in homozygosity (Table 4).

## 4. Discussion

This study investigated the registry-based incidence of DTD in Finland in subjects born between 1950 and 2020 and describes the clinical features of DTD in a non-selected group, including all Finnish DTD subjects born between 2000 and 2020. DTD has been the most prevalent skeletal dysplasia in Finland with reported disease incidence of 1:22,000 [11], compared to the estimated incidence of 1:100,000 in non-Finnish populations [9]. However, the incidence of DTD has not been recently explored. We noticed a significant decrease in the number of DTD subjects over the past decades. Ten children with DTD were born in 2000–2010 and only 4 children in 2010–2020. The decreased number of subjects with DTD is likely explained by increased prenatal diagnostics.

Our Finnish pediatric cohort comprised 14 subjects with *SLC26A2*-related skeletal dysplasia; 75% of all subjects and 90% of those with the DTD phenotype were homozygous for the Finnish founder mutation and one was (compound) heterozygous for the founder mutation. This is in line with previous reports [12], and characteristic of the diseases belonging to the ‘Finnish Disease Heritage’, caused by recessive founder mutations. The *SLC26A2* Finnish founder mutation is a splice-site mutation, usually found in homozygosity, which is classified as a severe mutation [12]. It causes DTD when homozygous, mild DTD/rMED in compound heterozygosity with mild alleles and ACG1B/AO2 in combination with severe alleles [36]. In our study, the patient with heterozygous Finnish founder mutation had a milder form of DTD, but information about the second variant was not available as genetic testing was performed elsewhere.

Two subjects with rMED phenotype had a combination of p.Arg279Trp and p.Thr512Lys. Their clinical and radiological findings have been described in detail previously [30,31]. These subjects presented with clinical features typical for rMED like mildly shortened stature, joint contractures, short limbs, clubfeet and hand deformities, but also cleft palate, cervical kyphosis and cystic swelling of the external ears. The p.Thr512Lys is a rare severe mutation, causing lethal form of dysplasia in homozygosity, DTD when compounded with the Finnish founder mutation and rMED when compounded with p.Arg279Trp. The p.Arg279Trp is a mild mutation reported to have considerable residual transporter activity and this milder mutation likely rescues the phenotype when compounded with the more severe mutation p.Thr512Lys [31,45].

The growth disturbance in DTD has its onset prenatally. Proteoglycans are important in the development and function of the growth plate. Animal studies have shown that slc26a2 has multiple roles in chondrocyte biology and that proteoglycan undersulfation causes decreased chondrocyte proliferation and lack of terminal differentiation. These factors have been suggested to contribute to reduced bone growth in DTD [2,4,46,47]. In our cohort, all children with DTD phenotype had a short stature. They were slightly shorter at birth compared to growth data from a large study from 1997 on 121 Finnish subjects with DTD, which reported a median birth length of 45.4 cm for boys and 45.0 cm for girls with DTD [14]. In our cohort head circumference at birth was normal. The growth failure in DTD is progressive with a first deviation during the first year [14]. This deviation was also seen in our study. In our pediatric cohort, median height SDS at last-follow-up was −5.5 for girls and −4.1 for boys with DTD (at median age of 12, and 13 years, respectively). We observed variability in the severity of growth failure among children with DTD with the same mutation. Other factors, like the degree of spine deformities and joint contractures can affect the height and these progressive clinical features can also account for part of the progressive growth failure [14]. The children with rMED had mildly shortened stature (−2.9 SD) at last follow-up.

The most common clinical features in this cohort, apart from short stature, were hand abnormalities. Other common clinical findings were cervical kyphosis, valgus deformity, lateral position of the patella, small chin and ACL absence/laxity; these manifestations were found in over 70% of the patients. Cleft palate, club foot, patellar luxation and lumbar lordosis were found in over half of the patients. The prevalence of cleft palate in our cohort (64%) was comparable to previous Finnish studies [48,49,50] and higher than the prevalence of 37.5% observed in classical DTD patients in a Portuguese cohort [13].

Spinal deformities are often associated with DTD [51]. Cervical kyphosis was highly prevalent in our cohort (79%). We observed varying severity of cervical kyphosis; in four subjects the kyphosis had resolved by the age of 7, one patient required operation and one child with very severe kyphosis deceased neonatally. This is in line with earlier studies showing that cervical kyphosis is common in childhood but tends to resolve with age [52,53]. Even severe forms of cervical kyphosis have been treated with beneficial and long-lasting results [54], but cervical kyphosis may in rare cases progress to extreme degrees, and cause spinal cord compression and death [52]. Scoliosis was present in 36% of our cohort. Previous studies have reported scoliosis in 37–90% of patients with DTD [51,55,56,57]. Scoliosis in DTD has shown variable natural history and can be classified into early-progressive, idiopathic-like and mild non-progressive [55]. Pronounced lumbar lordosis was found in over half of our study subjects. This prevalence was slightly higher than reported earlier [58]. Due to these significant spinal problems, the affected individuals should be carefully followed by a spine specialist.

Knee problems like early deformation, instability and flexion contractures are very common in DTD. The range of motion usually decreases already before the age of five and the knees show early degenerative changes from age six years onwards [59,60]. The children in our cohort showed high prevalence of valgus deformity and ACL absence/laxity, in line with previous studies [59,60]. Patellar luxation was seen in over half of the cohort. This is similar with a study that found patellar luxation in 63% of children with DTD [61].

More than one third of the children had respiratory problems as newborns. One child had a very severe form of DTD with severe cervical kyphosis, scoliosis and pulmonary hypoplasia leading to severe pulmonary hypertension and neonatal death. DTD has been associated with increased perinatal mortality, mainly because trachea- and broncho-malasia, and severe cervical kyphosis causing respiratory problems [53,58]. In the absence of severe complications in early childhood, individuals with DTD have good life expectancy [58].

An overlap between the phenotypes of the *SLC26A2* dysplasia family have been observed [13,30,31,35] and this was evident also in individual cases and cohorts described in the recent literature (Table 4). Some subjects with rMED have shown clinical manifestations that are more characteristic for DTD like cleft palate, auricular defects and severe cervical kyphosis [30,31,35]. The normal or mildly shortened stature seen in rMED can help to clinically distinguish it from DTD. We observed great variability in the severity of clinical manifestations among children with DTD with the same mutation, ranging from one very severe phenotype to milder forms of DTD. The severity of the clinical phenotype has been associated with the mutation type, the residual activity of the sulfate transporter and the extent of the under-sulfation of cartilage matrix [6] and some conclusions can be drawn also based on the recently reported cases (Table 4). However, the mutation type is not the only factor determining the phenotypic severity since clinical features also show intrafamilial variability. A large study reported that the severity and development of scoliosis in DTD cannot be predicted from genotype [57]. The phenotypic variability may be related to other genes, modifiers altering sulfate metabolism or environmental pre- and postnatal factors [13,57,62].

The diagnosis of DTD can usually be made prenatally or at birth. The majority of the children in our cohort were diagnosed with DTD prenatally and the remaining subjects were diagnosed at birth. Early prenatal diagnosis from DNA from chorionic villus is possible in subsequent pregnancies because of the 25% recurrence risk.

The main limitations of the study are the retrospective study design, the small cohort size and a short follow-up time for part of our pediatric cohort. Genetic data was absent or partial for some children as diagnosis was based on the typical clinical presentation only or genetic evaluation was performed elsewhere. Furthermore, because of the nature of our study, we were not able to obtain information regarding pregnancies that have been terminated in Finland during the past years because of suspected or confirmed DTD. However, it is likely that the decreasing incidence is partly explained by improved genetic counseling and increased prenatal diagnostics. In addition, increasing heterogeneity and mobility of the Finnish population may impact the incidence of the autosomal diseases caused by the old founder mutations.

The patient care in DTD requires a multi-disciplinary team in which especially orthopedic expertise is essential. Increased pathophysiological understanding and advances in the development of pharmacological treatments may hopefully offer individuals with DTD new therapeutic approaches in the future.

## 5. Conclusions

We observed a decreasing incidence of DTD in Finland, probably due to advanced prenatal diagnostics. The variable clinical severity, even in children with the same genotype, highlights the wide spectrum of clinical manifestations of *SLC26A2*-related disorders. The high phenotypic variability related to skeletal dysplasias can complicate the diagnosis. Genetic testing together with genetic counselling are important parts of the patient care.

## Figures and Tables

**Table 1:** Genetic information, clinical phenotype and growth data at birth and at 1 year and 5 years in the Finnish children.

Patient	*SLC26A2* Variant 1	*SLC26A2* Variant 2	Clinical Phenotype	Length at Birth (SDS)	Length at Age 1 (SDS)	Height at Age 5 (SDS)
1	N/A	N/A	DTD	−4.2	N/A	N/A
2	c.-26+2T>C	c.-26+2T>C	DTD	−4.0	−6.9	−5.6
3	c.-26+2T>C	c.-26+2T>C	DTD	−5.9	−7.4	−7.2
4	c.-26+2T>C	c.-26+2T>C	DTD	−5.3	−7.6	−5.4
5	c.-26+2T>C	c.-26+2T>C	DTD	−2.9	−6.5	−5.1
6	c.-26+2T>C	c.-26+2T>C	DTD	−3.5	−5.4	−4.7
7	c.-26+2T>C	c.-26+2T>C	DTD	−3.7	−4.5	N/A
8 *	c.-26+2T>C	c.-26+2T>C	DTD	N/A	N/A	N/A
9	c.-26+2T>C	c.-26+2T>C	DTD	−3.5	−5.3	−2.9
10 *	c.-26+2T>C	c.-26+2T>C	DTD	−6.3	−9.2	−7.9
11	N/A	N/A	DTD	−4.4	−6.2	N/A
12	c.-26+2T>C	N/A	DTD	N/A	N/A	−4.0
13	Arg279Trp	Thr512Lys	rMED	−2.2	−2.6	−2.9
14	Arg279Trp	Thr512Lys	rMED	−1.1	−0.3	N/A

N/A = not available; * siblings.

**Table 2:** Growth data at birth for children affected with DTD and rMED. Data presented as median (range).

	Boys	Girls
**DTD**	n = 4	n = 6
Length (cm)	42.5 (39–44.5)	43 (39.5–45)
Weight (g)	3310 (2615–3570)	3390 (2525–3900)
Head circumference (cm)	36.5 (35–37)	35.5 (32–38)
**rMED**	n = 2	n = 0
Length (cm)	48 (47–49)	
Weight (g)	3680 (3230–4120)	
Head circumference (cm)	36 (35–37)	

**Table 3:** Prevalence of clinical features in the Finnish children with *SLC26A2*-related skeletal dysplasia. Values are given for the whole cohort and separately for children with DTD and rMED.

Clinical Features	All (n = 14)	DTD (n = 12)	rMED (n = 2)
Hand abnormalities	100%	100%	100%
Cleft palate	64%	67%	50%
Naevus flammeus	21%	25%	0%
Small chin	71%	75%	50%
Auricular abnormality	36%	33%	50%
Club foot	57%	58%	50%
Other foot deformity	21%	17%	50%
ACL absence/laxity	71%	75%	50%
Lateral position of patella	79%	83%	50%
Patellar luxation	57%	58%	50%
Valgus deformity	86%	83%	100%
Cervical kyphosis	79%	83%	50%
Scoliosis	36%	33%	50%
Lumbar lordosis	57%	58%	50%

**Table 4:** Genetic and clinical findings in previously reported non-Finnish subjects and cohorts with *SLC26A2*-related DTD and rMED. Empty spaces indicate that detailed data were not available for the specific feature.

*SLC26A2* Variant 1	*SLC26A2* Variant 2	Phenotype	n	Brachy-dactyly	Other Hand Deformities	Cleft Palate	Auricular Abnormality	Club Foot	Other Foot Deformity	Valgus Deformity	Patellar Luxation	Cervical Kyphosis	Scoliosis	Lumbar Lordosis	Reported in
Arg279Trp	Arg279Trp	rMED	27	15%	30%	7%	4%	41%	7%	4%			4%	7%	[13,17,32,33]
Arg279Trp	Arg178Ter	DTD	8	88%	100%	25%	100%	88%	13%			88%	63%	38%	[13,34]
c.-26+2T>C	Arg279Trp	rMED	4		25%	25%	25%	0%	25%	25%		50%	75%		[35]
c.-26+2T>C	Arg279Trp	Intermediate	4	75%	75%	50%	50%	50%	25%	50%		25%	25%		[36,37]
Cys653Ser	Cys653Ser	rMED	6		83%				17%	17%	50%		50%		[18,38,39]
Cys653Ser	Ala715Val	Intermediate	3	100%	100%	0%	0%	67%	33%	33%	67%		67%		[40]
Arg279Trp	c.727-1G>C	Intermediate	2	100%	100%	0%	100%	100%				100%	50%	50%	[13]
Leu275Pro	Leu400Phe	rMED	2	50%		0%			50%				50%		[41]
Val162fs	Asp385Asn	rMED	1	100%		0%	0%		100%	100%		0%	0%	0%	[42]
c.-26+2T>C	c.-26+2T>C	DTD	1			100%	100%	100%					100%		[35]
Arg279Trp	Asn425Asp	DTD	1	100%	100%	100%	100%	100%				100%	100%	100%	[13]
Arg279Trp	Ser522Phe	rMED	1	100%		0%			100%	100%					[43]
Thr266Ile	Val340del	Intermediate	1		100%			100%					100%		[44]
Arg279Trp	Thr512Lys	rMED	1			0%	100%	100%				0%	0%	0%	[35]

## Data Availability

The data presented in this study are available on request from the corresponding author. The data are not publicly available due to ethical reasons.
